# PTP1B Modulates Carotid Plaque Vulnerability in Atherosclerosis Through Rab5‐PDGFRβ‐Mediated Endocytosis Disruption and Apoptosis

**DOI:** 10.1111/cns.70071

**Published:** 2024-11-08

**Authors:** Xiao Zhang, Ran Xu, Tao Wang, Jiayao Li, Yixin Sun, Shengyan Cui, Zixuan Xing, Xintao Lyu, Ge Yang, Liqun Jiao, Wenjing Li

**Affiliations:** ^1^ Department of Neurosurgery Xuanwu Hospital, Capital Medical University Beijing China; ^2^ China International Neuroscience Institute (China‐INI) Beijing China; ^3^ First Hospital Peking University Beijing China; ^4^ Health Science Center Peking University Beijing China; ^5^ Health Science Center Xi'an Jiaotong University Shanxi China; ^6^ Capital Medical University Beijing China; ^7^ Laboratory of Computational Biology and Machine Intelligence, National Laboratory of Pattern Recognition, Institute of Automation Chinese Academy of Sciences Beijing China; ^8^ School of Artificial Intelligence University of Chinese Academy of Sciences Beijing China; ^9^ Department of Interventional Neuroradiology Xuanwu Hospital, Capital Medical University Beijing China

**Keywords:** apoptosis, atherosclerosis, carotid plaque, endocytosis disruption, PTP1B

## Abstract

**Background:**

Protein tyrosine phosphatase 1B (PTP1B) is a protein tyrosine phosphatase and modulates platelet‐derived growth factor (PDGF)/platelet‐derived growth factor receptor (PDGFR) signaling in vascular smooth muscle cells (VSMCs) via endocytosis. However, the related molecular pathways that participated in the interaction of endo‐lysosome and the trafficking of PDGFR are largely unknown. This study aims to determine the subcellular regulating mechanism of PTP1B to the endo‐lysosome degradation of PDGFR in atherosclerotic carotid plaques, thereby offering a potential therapeutic target for the stabilization of carotid plaques.

**Methods:**

The immunohistochemical staining technique was employed to assess the expression levels of both PDGFR‐β and Caspase 3 in stable and vulnerable carotid plaques. Tunnel staining was utilized to quantify the apoptosis of carotid plaques. Live‐cell imaging was employed to observe endocytic motility, while cell apoptosis was evaluated through Propidium Iodide staining. In an in vivo experiment, ApoE^−/−^ mice were administered a PTP1B inhibitor to investigate the impact of PTP1B on atherosclerosis.

**Results:**

The heightened expression of PDGFR‐β correlates with apoptosis in patients with vulnerable carotid plaques. At the subcellular level of VSMCs, PDGFR‐β plays a pivotal role in sustaining a balanced endocytosis system motility, regulated by the expression of Rab5, a key regulator of endocytic motility. And PTP1B modulates PDGFR‐β signaling via Rab5‐mediated endocytosis. Additionally, disrupted endocytic motility influences the interplay between endosomes and lysosomes, which is crucial for controlling PDGFR‐β trafficking. Elevated PTP1B expression induces cellular apoptosis and impedes migration and proliferation of carotid VSMCs. Ultimately, mice with PTP1B deficiency exhibit a reduction in atherosclerosis.

**Conclusion:**

Our results illustrate that PTP1B induces disruption in endocytosis and apoptosis of VSMCs through the Rab5‐PDGFRβ pathway, suggesting a potential association with the heightened vulnerability of carotid plaques.

## Graphical abstract

This study illustrates that PTP1B induces disruption in endocytosis and apoptosis of VSMCs through the Rab5‐PDGFRβ pathway, suggesting a potential association with the heightened vulnerability of carotid plaques. Our results also present novel evidence supporting the potential use of PTP1B inhibitors in the treatment of vulnerable carotid plaques.

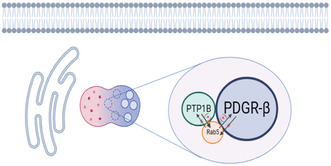

## 1 Introduction

Stroke, the second leading cause of death worldwide as well as the leading cause in China, is a heavy burden in modern society [1]. The majority of strokes are ischemic strokes. As a well‐recognized risk factor, carotid artery stenosis accounts for up to 20% of ischemic strokes [2]. In a community‐based investigation of patients with asymptomatic severe carotid stenosis who did not receive surgeries, the estimated rate of ipsilateral carotid‐related acute ischemic stroke was 4.7% over 5 years [3]. In this line, patient risk stratification should be emphasized, which is mainly depends on the extent of stenosis and plaque vulnerability. Increasing evidence has indicated that unstable plaques in the carotid arteries are more prone to initiate embolization, regardless of the degree of stenosis [2]. Thus, a deeper understanding on underlying mechanisms in effectors of plaque vulnerability is critical for clinical management of carotid artery stenosis.

Vulnerable plaques, featured by a large necrotic core with a high content of lipids and inflammatory cells, are covered by a thin fibrous cap, whereas stable plaques are represented with thick fibrous caps with vascular smooth muscle cells (VSMCs) producing extracellular matrix [4]. In fact, VSMCs are the commonest cell types involved in the vulnerability of carotid atherosclerotic plaques, through phenotype switch from contractile phenotype to secretory or even macrophage‐like phenotypes, which has been considered as the main approach to increase plaque vulnerability [5]. The function and fate of VSMCs can be reflected and mediated by multiple subcellular processes, especially the interaction between various organelles. Inhibition of lysosomal and endosomal function facilitated osteogenic transformation of VSMCs, which acted as a promoter of atherosclerotic vascular calcification [6]. In addition, acetylated LDL/cholesterol‐loaded lysosomes could be transported from macrophages into VSMCs [7]. The lysosomal transport led to a phenotypic switch of the VSMCs toward a foam cell‐like cell phenotype, which was suspected to reduce plaque stabilizing properties of VSMCs [7]. Nevertheless, the targets and molecular mechanisms of endo‐lysosome‐mediated plaque stabilizing remain to be clarified.

Platelet‐derived growth factor receptor (PDGFR) belongs to receptor tyrosine kinases (RTKs), which play fundamental roles in normal growth, development, tumorigenesis, and its therapeutic targeting [8]. There are two recognized members of PDGFR, PDGFR‐α and PDGFR‐β. The PDGF signaling system is activated by binding between PDGFR and the ligands, including PDGFA, ‐B, ‐C, and ‐D. In previous studies, PDGF and PDGFRs were found to be highly expressed in atherosclerotic vessels compared with normal controls [9, 10]. Furthermore, PDGFRβ was activated in VSMCs, which resulted in increased secretion of chemokines and leukocyte recruitment, and therefore promoted plaque formation [11]. The elevated biological behaviors of VSMCs by activation of PDGF can be attenuated by protein tyrosine phosphatase 1B (PTP1B) [12]. In fact, PTP1B was identified as protein tyrosine phosphatase associating with the PDGF receptor two decades ago [13]. Theoretically, PTP1B can modulate PFGF/PDGFR signaling in VSMCs via endocytosis [14]. However, the related molecular pathway that participated in the interaction of endo‐lysosome and the trafficking of PDGFR is largely unknown.

In this study, we investigated the expression pattern of PTP1B in atherosclerotic carotid plaques and clarified the subcellular regulating mechanism of PTP1B to the endo‐lysosome degradation of PDGFR, through histological and biological assays. We found that elevated expression of PTP1B disrupted the interaction of endo‐lysosome, which resulted in the accumulation of PDGFR, and therefore promoted plaque vulnerability. Our study may provide PTP1B as a potential therapeutic target in stabilizing carotid plaque at a subcellular level.

## 2 Methods

### 2.1 Ethics Statement

This study received approval from the Ethics Committee of Xuanwu Hospital, Capital Medical University, under the reference number KS2021124‐1. The human research carried out in this study adhered to the principles outlined in the Declaration of Helsinki (revision 6, 2008) and was conducted in accordance with institutional guidelines. Informed consent was obtained from all patients who underwent carotid endarterectomy and contributed specimens to this study.

### 2.2 Specimen Collection

Carotid plaques were collected from individuals with either asymptomatic or symptomatic high‐grade carotid artery stenosis (> 50% for symptomatic patients and > 70% for asymptomatic patients, following the criteria of the North American Symptomatic Carotid Endarterectomy Trial for carotid stenosis) at Xuanwu Hospital, Capital Medical University. The plaques were categorized into stable and vulnerable types based on ultrasound and histopathological assessments. Vulnerable plaques exhibited features such as intraplaque hemorrhage, mural thrombus, thin fibrous caps, or an incomplete fibrous cap, while those lacking these characteristics were classified as stable plaques.

### 2.3 Experimental Animals

Animal protocols were approved by the Animal Care and Use Committee of Capital Medical University, and the study was performed according to the National Institutes of Health Guide for the Care and Use of Laboratory Animals. Eight‐week‐old female mice of wild‐type and ApoE^−/−^ mice on a C57BL/6 background were obtained from SPF Biotechnology (Beijing, China). ApoE^−/−^ mice were randomly enrolled into two groups: vehicle and KY226 (HY‐120327, MedChem Express, 10 mg/kg per day). All the mice were fed with a high‐fat diet (0.15% cholesterol and 21% fat, 4 kcal/g) during the 12‐week experiment. Mice were anesthetized with 1% pentobarbital sodium (60 mg/kg) followed by anatomical neck tissue. Then the connected fat tissues were removed and the common carotid arteries were individualized. The bilateral common carotid arteries were harvested and stored in 4% paraformaldehyde for histological staining and immunohistochemistry.

### 2.4 Cell Culture and Transfection

Human carotid vascular smooth muscle cells (VSMCs) were procured from Bluefbio (Shanghai, China) and cultured in Dulbecco modified Eagle medium (DMEM; Life Technologies, USA), supplemented with 10% fetal bovine serum (FBS; Thermo Fisher Scientific, USA). The cells were maintained in a 5% CO_2_ incubator at 37°C. Lentiviruses for knockdown or overexpression of human PTP1B, PDGFR‐β, and Rab5 were constructed by GeneChem (Shanghai, China). The sequences of lentiviruses were listed as follows: sh‐PTPNI 5′‐GGAAGAGACCCAGGAGGATAA‐3′; sh‐Rab5 5′‐GGCAAGCAAGTCCTAACATTG‐3′; sh‐PDGFR‐β 5′‐CCGGGCTCACCATCATCTCCCTTATCTCGAGATAAGGGAGATGATGGTGAGCTTTTT‐3′. Lentiviral transfection was performed following previously described methods and the manufacturer's protocol [15]. Lentiviral transfection was initiated when cells reached 20%–30% confluence. Based on the titer determination results, knockdown lentivirus or control lentivirus was added to the medium at the appropriate multiplicity of infection and incubated for approximately 24 h. Following this, the medium was replaced with a normal complete medium supplemented with puromycin (ST551, Beyotime, Shanghai, China) to establish stable cell populations for subsequent experiments. The efficiency of lentiviral transfection was confirmed using western blot analysis. Inhibitors were obtained from MedChem Express, including Sitravatinib (HY‐16961) and CP673451 (HY‐12050), which were used as inhibitors for PDGFR.

### 2.5 Live Cell Imaging

In the context of live cell imaging, cells labeled with fluorescent markers were cultured on glass‐bottomed MatTek dishes until reaching 70% confluency. Subsequently, the imaging process utilized a 100× 1.45NA oil immersion objective mounted on an Eclipse Ti2‐E inverted microscope (Nikon). The imaging system incorporated a CSUW1 Spinning Disk scanning head (Yokogawa) and a Prime 95B sCMOS camera (Photometrics), all meticulously orchestrated through the Nikon Elements software. To maintain cellular conditions, a constant temperature of 37°C with 5% CO_2_ was upheld within an on‐stage incubator provided by Tokai Hit. During the imaging sessions, cell staining markers associated with endosomes and lysosomes were captured within a single focal plane for a duration of 2 min, with images acquired at 2‐s intervals. For the purpose of single particle tracking, images were consistently captured at 2‐s intervals to achieve optimal temporal resolution.

### 2.6 Tracking and Categorization of Particles

The trajectories of endosomes or lysosomes in time‐lapse videos were acquired as CSV files utilizing the TrackMate plugin within the FIJI software. Subsequently, our custom MATLAB program (The MathWorks Inc., 2017) was employed for analysis. The primary objective was to calculate the mean square displacement (MSD) for each trajectory and subsequently categorize the particles into either confined or directed movement patterns. For particles exhibiting a duration of more than five frames, the MSD was computed using the MSD analyzer, with a maximum lag of 20 frames.

### 2.7 Histopathological Examination

Carotid plaques underwent fixation with 4% paraformaldehyde, followed by dehydration using gradient ethanol, transparency with xylene, and were embedded in paraffin. Subsequently, the fixed plaques were sectioned in a tissue slicer at a thickness of 4 μm. The sections were then stained with hematoxylin and eosin (HE) and Masson's trichrome, following standard protocols. Immunohistochemical staining was conducted using anti‐PTP1B (11334‐1‐AP; Proteintech), anti‐PDGFR‐β (13499‐1‐AP; Proteintech), anti‐Rab5 (11947‐1‐AP; Proteintech), and anti‐Cleaved Caspase‐3 (25128‐1‐AP; Proteintech), following previously reported protocols.

### 2.8 Evaluation of Apoptosis

Apoptotic cells were identified by staining with propidium iodide (PI) staining solution (Cell Signaling Technology), while nuclei were stained with Hoechst 33342 (Cell Signaling Technology). The cells were incubated at 37°C with 5% CO_2_ in an on‐stage incubator (Tokai Hit, USA) during imaging. For each well, 20 randomly selected fields of view were captured and analyzed using ImageJ software to determine the proportion of apoptotic cells. Additionally, carotid plaque apoptosis was assessed through TUNEL staining. Paraffin sections of carotid plaques were deparaffinized with xylene and dehydrated using a graded ethanol series. Subsequently, the sections were incubated with TUNEL staining solution (Abcam, USA) at 37°C for 1 h and observed using a fluorescent microscope.

### 2.9 Western Blotting

Proteins were extracted using RIPA lysis Buffer (Abcam, Ab156034, UK), followed by centrifugation at 12,000 *g* for 15 min at 4°C. Protein concentrations were determined using the BCA method. A total of 20 μg of cell proteins were loaded onto a 4%–12% polyacrylamide gel and separated by electrophoresis. Subsequently, proteins were transferred to polyvinylidene fluoride membranes, blocked in 5% skim milk for 0.5 h, and incubated at 4°C overnight with the following primary antibodies: anti‐PTP1B (11334‐1‐AP; Proteintech), anti‐GAPDH (60004‐1‐AP; Proteintech), anti‐PDGFR‐β (13499‐1‐AP; Proteintech), anti‐Rab5 (11947‐1‐AP; Proteintech), and anti‐Cleaved Caspase‐3 (25128‐1‐AP; Proteintech). The secondary antibodies (1:10,000) were incubated with the membranes for 1 h at room temperature. Blots were visualized using the ECL chemiluminescent substrate reagent kit. For quantitative analysis of Western blotting, Fuji software was utilized to measure the average gray value of the bands, obtaining a standardized ratio compared to the gray value of the bands in the internal reference. Statistical analysis was then performed based on the magnitude of the ratio.

### 2.10 EdU Stain Assay

Carotid VSMCs were cultured in DMEM medium and incubated with a 50 μmol/L EdU solution (C10086; Invitrogen, USA) for 2 h. Following incubation, the cells were fixed with 4% paraformaldehyde for 30 min and treated with 2 mg/mL glycine for 5 min. Subsequently, cells were washed three times with PBST for 10 min each. The Apollo reaction solution was added to the cells, and nuclei were stained with Hoechst 33342 for 30 min at room temperature. EdU‐positive cells were visualized and detected using a fluorescence microscope.

### 2.11 Cell Scratch Wound Assay

Carotid VSMCs were plated in a six‐well plate and subjected to transfection and treatment. After 24 h, a straight line was carefully drawn across the plate using a 1000‐μL pipette tip. Subsequently, the medium was replaced with serum‐free medium for additional incubation. Images of the cells within the scratch were captured at 0 h and 12 h using a microscope. The migration ability of the cells was analyzed based on the extent of healing observed in the scratched area.

### 2.12 Statistical Analysis

Data are expressed as the mean ± standard deviation (SD). Continuous variables were assessed for normality using the one‐sample Kolmogorov–Smirnov test. The independent samples t‐test was employed for continuous variables that followed a normal distribution. Data that did not exhibit a normal/Gaussian distribution were analyzed using a nonparametric equivalent. Statistical analysis was performed using GraphPad Prism version 9.00 software (GraphPad). *p* values < 0.05 were considered statistically significant.

## 3 Results

### 3.1 The Accumulation of PDGFR‐β Expression Leads to Apoptosis in Patients With Vulnerable Carotid Plaques

**FIGURE 1:**
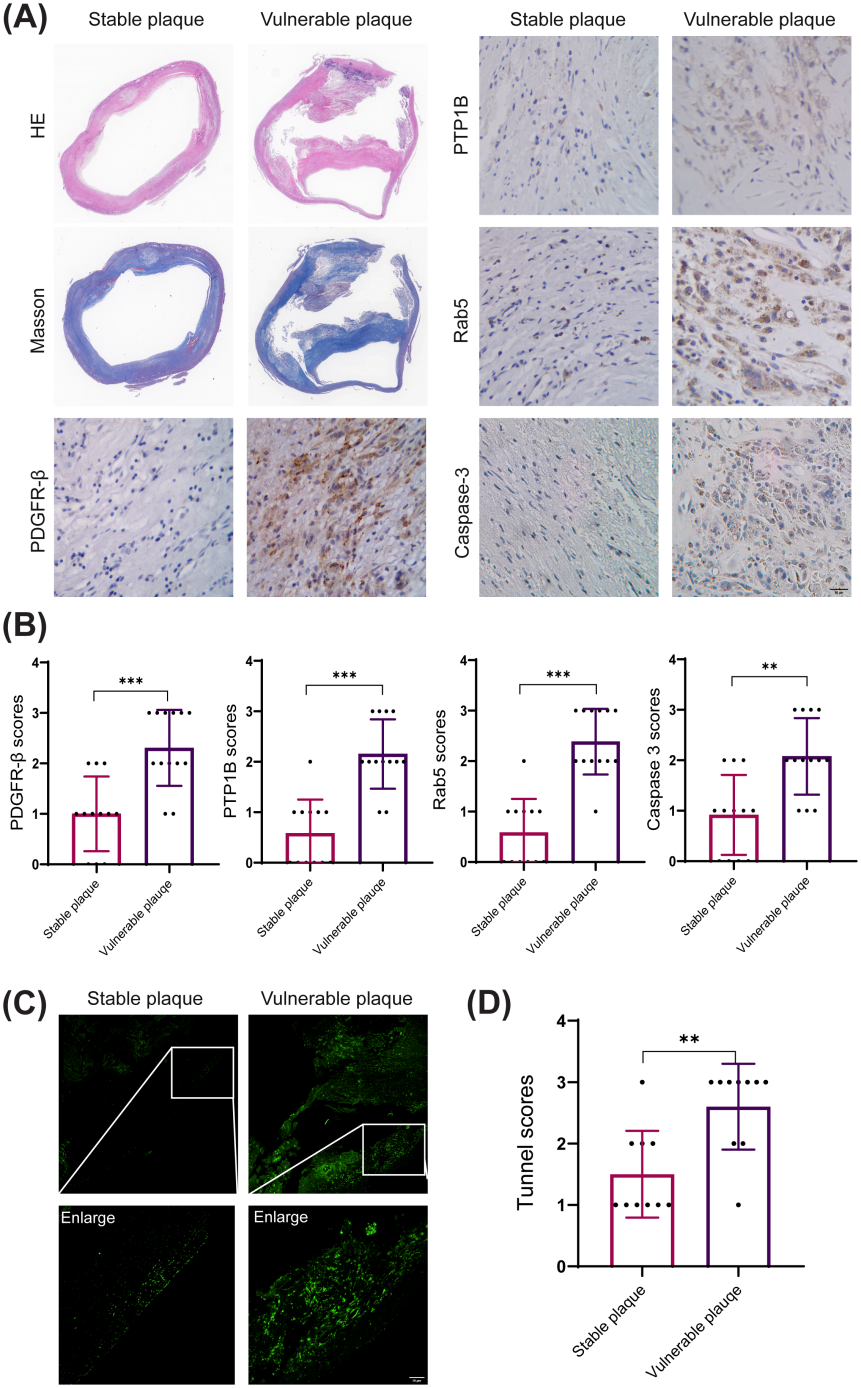
The accumulation of PDGFR‐β expression leads to apoptosis in patients with vulnerable carotid plaques. HE, Hematoxylin–Eosin. (A) HE, Masson, and immunohistochemical staining for PDGFR‐β, PTP1B, Rab5, and Caspase‐3 in representative stable plaques and vulnerable carotid plaques. (B) Quantitative analysis of immunohistochemical staining scores for PDGFR‐β, PTP1B, Rab5, and Caspase‐3 in stable and vulnerable carotid plaques. (C) Tunnel staining for representative stable plaques and vulnerable carotid plaques. (D) Quantitative analysis of tunnel staining score for stable and vulnerable carotid plaques. Scar bar 10 μm. **p < 0.01, ***p < 0.001.

The apoptosis of atherosclerotic plaque induces several features of plaque vulnerability, including fibrous cap thinning, increased necrotic cores, and inflammatory factors [16]. In order to detect the relationship between PDGFR‐β and carotid plaque vulnerability, 25 carotid artery plaques (12 stable plaques and 13 vulnerable plaques) were conducted pathological test (Figure 1A). Immunohistochemistry of PDGFR‐β and Caspase‐3 was performed, and the results showed higher expressions of PDGFR‐β and Caspase‐3 in vulnerable plaque compared to stable plaque (Figure 1A,B). To investigate the relationship between PTP1B and Rab5 expression levels and plaque vulnerability, we also utilized immunohistochemistry to detect the expression levels of PTP1B and Rab5. The results revealed that the expressions of PTP1B and Rab5 were significantly elevated in vulnerable plaques compared to stable plaques (Figure 1A,B). The level of plaque apoptosis was also examined by Tunnel stain, and the results showed that apoptosis was markedly aggravated in vulnerable plaque (Figure 1C,D). Overall, these results suggest that the accumulation of PDGFR‐β is associated with plaque apoptosis, which potentially leads to carotid plaque vulnerability.

### 3.2 PDGFR Is Required for Sustained and Balanced Motility of the Endocytosis System in Vascular Smooth Muscle Cells

**FIGURE 2:**
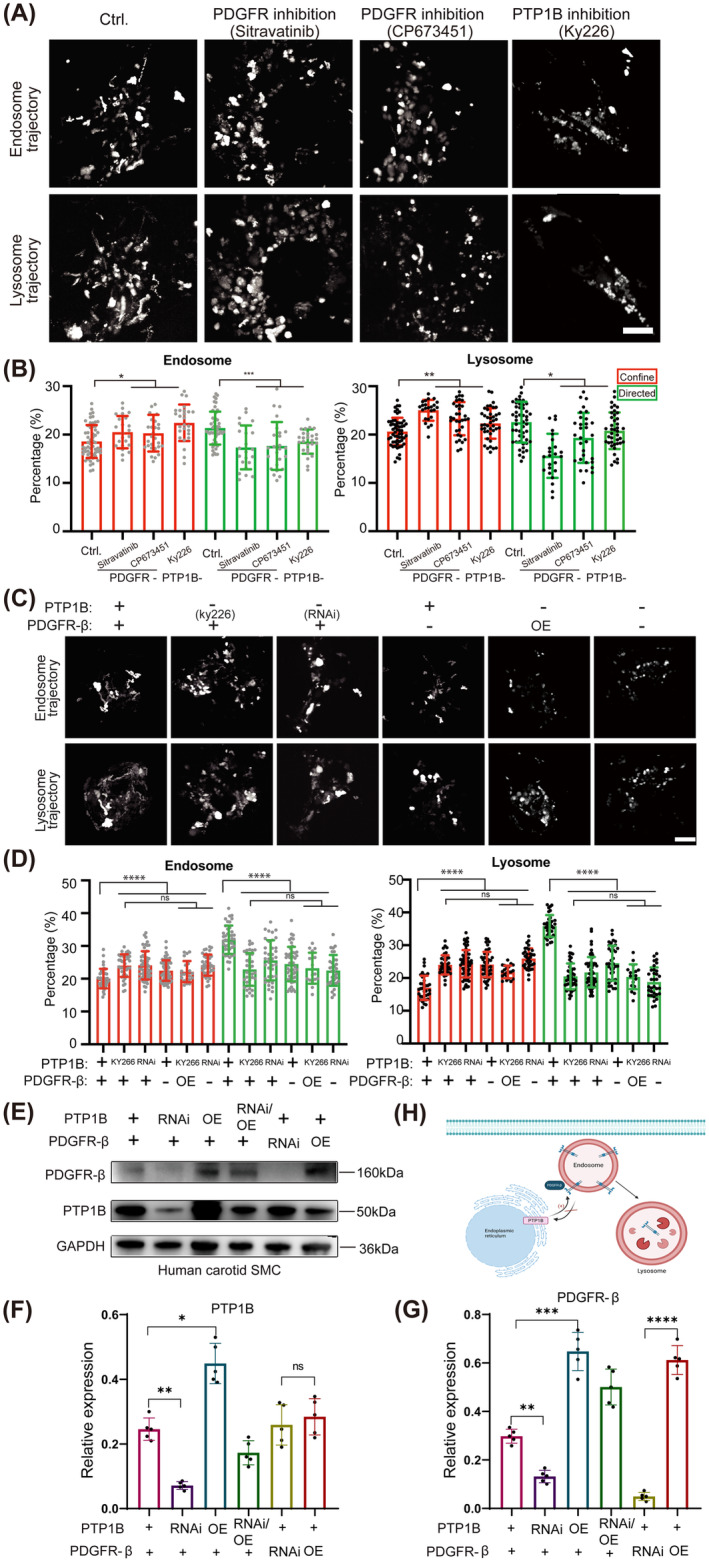
PDGFR is required for sustained and balanced motility of the endocytosis system in vascular smooth muscle cells. Ctrl, Control; OE, Overexpression; RNAi, RNA interference. (A) Endosome and lysosome trajectories of carotid VSMCs with the treatment of PDGFR inhibitors (Sitravatinib and CP673452), and PTP1B inhibitor (KY226). (B) Quantitative analysis of confined and directed modes of endosome and lysosome. (C) Endosome and lysosome trajectories of cos7 cells with the treatments of PDGFR‐β inhibitor and overexpression, and PTP1B inhibitor or RNA knockdown. (D) Quantitative analysis of confined and directed modes of endosome and lysosome. (E) Western blot analyses of PDGFR‐β and PTP1B. (F) The relative level of PTP1B/GAPDH (*n* = 5). (G) The relative level of PDGFR‐β/GAPDH (*n* = 5). (H) Schematic diagram of the relationship between PTP1B and PDGFR‐β on endocytosis system. Scar bar 10 μm. *p < 0.05, **p < 0.01, ***p < 0.001, ****p < 0.0001, and ^ns^ *p* > 0.05.

It is well characterized that the intracellular recycling of platelet‐derived growth factor receptors (PDGFR) is primarily regulated by the endocytic pathway. PDGFR is internalized into early endosomes, traffics via mobile endosomes, and is delivered to multivesicular bodies (MVBs) /lysosomes or recycled back to the plasma membrane. Owing to its importance as an activity regulator, sustained and balanced motility of endocytosis is critical for the trafficking, degradation, and activity of PDGFR (The emerging complexity of PDGFRs: activation, internalization, and signal attenuation). To investigate whether PDGFR‐β is associated with the altered dynamics of endocytosis, we analyzed the motility of both endosomes and lysosomes at the whole‐cell scale in live vascular smooth muscle cells (VSMCs). We labeled endosomes and lysosomes using dextran Alexa Fluor 647 and 561, respectively (Endo‐lysosomal vesicles positive for Rab7 and LAMP1 are terminal vesicles for the transport of dextran) and imaged every 2 s for 3 min. For each single cell, we tracked individual endosomes and lysosomes using the Trackmate software from Fiji (TrackMate: an open and extensible platform for single‐particle tracking) and analyzed their dynamic behavior by mean square displacement (MSD) analysis, in which particles are subdivided into confined, directed, or uncharacterized modes based on their motility. Confined mode represents the status where particles rarely move, with a diffusive parameter less than 0.9, and their trajectories appear as dots, whereas the diffusive parameter of directed particles is > 1.2, showing the linear trajectories (Figure 2A). We applied two PDGFR inhibitors Sitravatinib (1 μM) and CP673451 (1 μM) to cells and found increased constrained diffusion (20.5% and 20.2%) and decreased directed diffusion (17.7% and 17.4%) of endosomes compared with control cells (18.6% confined and 21.3% directed). Consistent changes in lysosome dynamics were also found, the confined proportion was increased to 25.0% and 23.3% compared with 20.6% in control, and directed particles were reduced to 15.6% and 19.3% compared with 22.6% in control (Figure 2A,B).

To further investigate how PDGFR and PTP1B regulate endocytosis, we first compared the motility of endosomes and lysosomes in cos7 cells that express very low levels of endogenous PTP1B through RNA interference (RNAi) or show low kinase activity by an inhibitor (Figure 2C). Both reduced expression or activity of PTP1B results in less dynamic endocytosis. Confined endosomes were increased from ~20% (20.0%–24.0%/24.1%, respectively), and moving endosomes were reduced by ~20% (31.9%–25.6% and 22.9%, respectively). The subpopulation of lysosomes was similarly changed, confined lysosomes increased by 18% (from 17.0% to 24.0%/24.4%), and directed lysosomes were reduced by 40% (from 35.9% to 21.7%/20.5%) (Figure 2D). Consistently, PDGFR inhibition blocks the movement of endosomes/lysosomes, confined endosomes or lysosomes were increased by ~10%, and dynamic particles were reduced by 20% (Figure 2D). Additionally, overexpressing of PDGFR slightly rescued the slowed‐down endocytosis resulting from PTP1B inhibition (Figure 2D). Further overexpressing PDGFR or inhibition of PDGFR changed dynamics by less than 4% compared to PTP1B inhibition. The slight recovery or enhancement of slowed‐down endocytosis resulting from PTP1B inhibition (Figure 2D), suggesting that PDGFR regulates the motility of endocytosis mainly through PTP1B activity.

In parallel, we established the knockdown and overexpression of PDGFR and PTP1B in VSMCs. The results showed that the expression of PDGFR‐β was significantly enhanced or decreased by overexpression or knockdown of PTP1B, respectively. However, the expression of PTP1B was not affected by overexpression or knockdown of PDGFR‐β (Figure 2E–G). The results demonstrated that the expression of PDGFR‐β was also regulated by PTP1B (Figure 2H).

### 3.3 Loss of PDGFR Decreases the Expression of Rab5 Which Is Responsible for the Endocytic Motility

**FIGURE 3:**
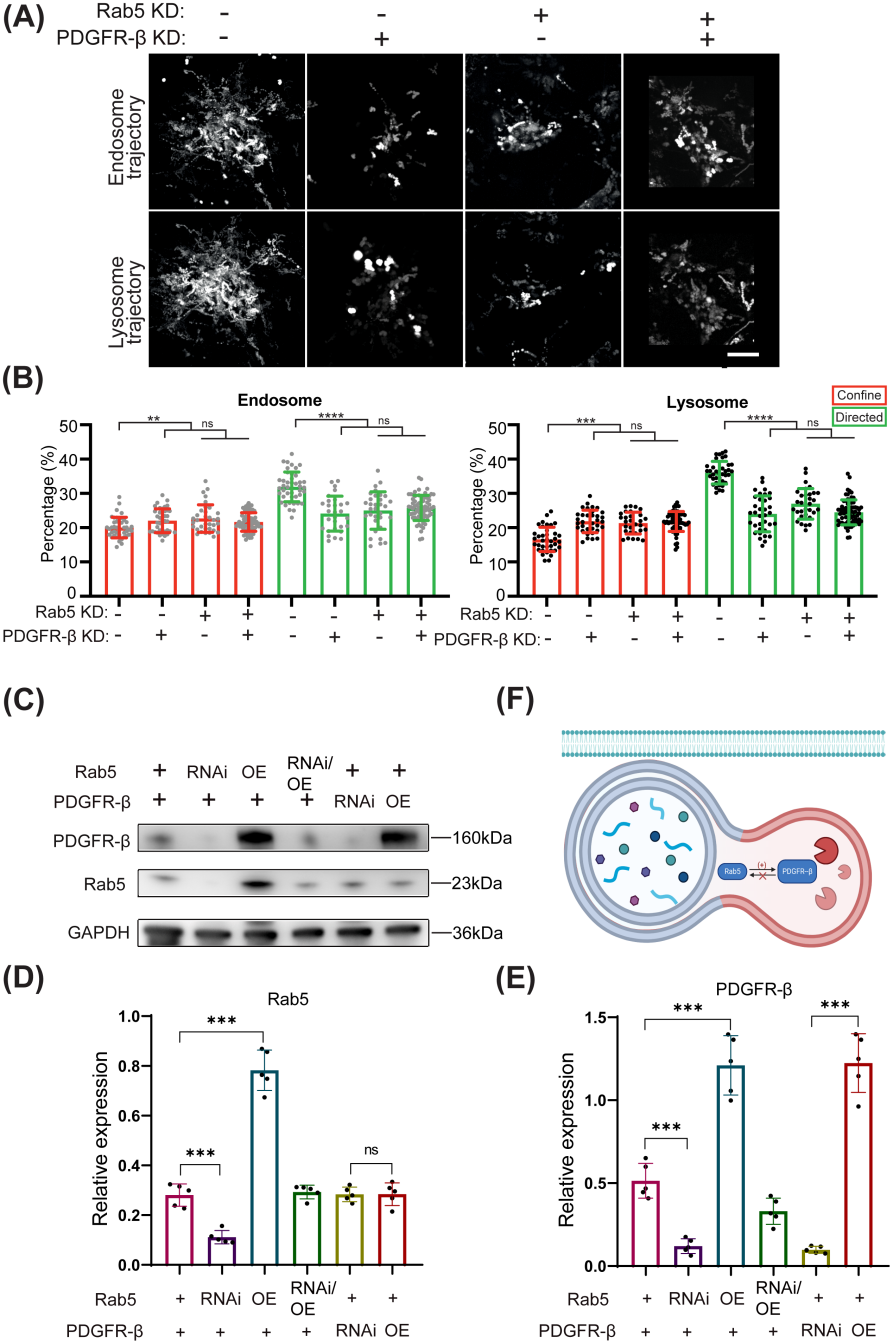
Loss of PDGFR decreases the expression of Rab5 which is responsible for the endocytic motility. KD, Knockdown; OE, Overexpression; RNAi, RNA interference. (A) Endosome and lysosome trajectories of cos7 cells with the treatments of Rab5 knockdown, PDGFR‐β knockdown, or both knockdowns. (B) Quantitative analysis of confined and directed modes of endosome and lysosome. (C) Western blot analyses of PDGFR‐β and Rab5. (D) The relative level of Rab5/GAPDH (*n* = 5). (E) The relative level of PDGFR‐β/GAPDH (*n* = 5). (F) Schematic diagram of the relationship between Rab5 and PDGFR‐β on endocytosis system. Scar bar 10 μm. **p < 0.01, ***p < 0.001, ****p < 0.0001, and ^ns^ *p* > 0.05.

Rab proteins serve an important role in cargo selection and trafficking effector recruitment. Deregulation of Rab5 and Rab4 proteins in p85R274A‐expressing cells alters PDGFR trafficking. As one of the best characterized Rab proteins, Rab5 shows the activity on mediating the PDGFR internalization from the plasma membrane to the early endosome and the sorting of PDGFR into multivesicular bodies destined for lysosomal degradation. Rab5 is a signaling GTPase involved in actin remodeling by receptor tyrosine kinases. Since the evidence for dysregulated PTP1B‐PDGFR signaling in both VSMCs and Cos7 cells primarily affects the endocytic dynamics, we next examined whether Rab5, whose activity is regulated by PDGFR‐β, modulates endocytic trafficking. We have generated two independent Rab5 shRNA expressing cos7 lines, and the substantial disruption of endocytosis particles' movement was observed (Figure 3A). Rab5 RNAi phenocopies the globally shifted endo‐lysosomal dynamics resulted from PDGFR‐β inhibition (10%~32% increased confined endosome/lysosomes and 24%~33% decreased directed endosome/lysosomes), showing 13%~22% increment of confined endosome/lysosomes and 27%~34% reduction of directed endosome/lysosomes (Figure 3B). Double knockout of PDGFR‐β and Rab5 shows a similar level of endosome trafficking reduction compared with PDGFR‐β inhibition or Rab5 RNAi and leads to 8%~32% increased confined endosome/lysosomes and 19%~33% decreased directed endosome/lysosomes (Figure 3B). The recapitulated phenotype of less dynamic endocytosis suggests that the reduction of PDGFR impairs Rab5‐dependent endo‐lysosomal trafficking.

Accordingly, we established the knockdown and overexpression of Rab5 and PDGFR‐β in cos7 lines. The results showed that the expression of PDGFR‐β was significantly enhanced or decreased by overexpression or knockdown of Rab5, respectively. However, the expression of Rab5 was not affected by overexpression or knockdown of PDGFR‐β (Figure 3C–E). The results demonstrated that the expression of PDGFR‐β was also regulated by Rab5 (Figure 3F).

### 3.4 PTP1B Modulates PDGFR‐β Signaling via Rab5‐Mediated Endocytosis

**FIGURE 4:**
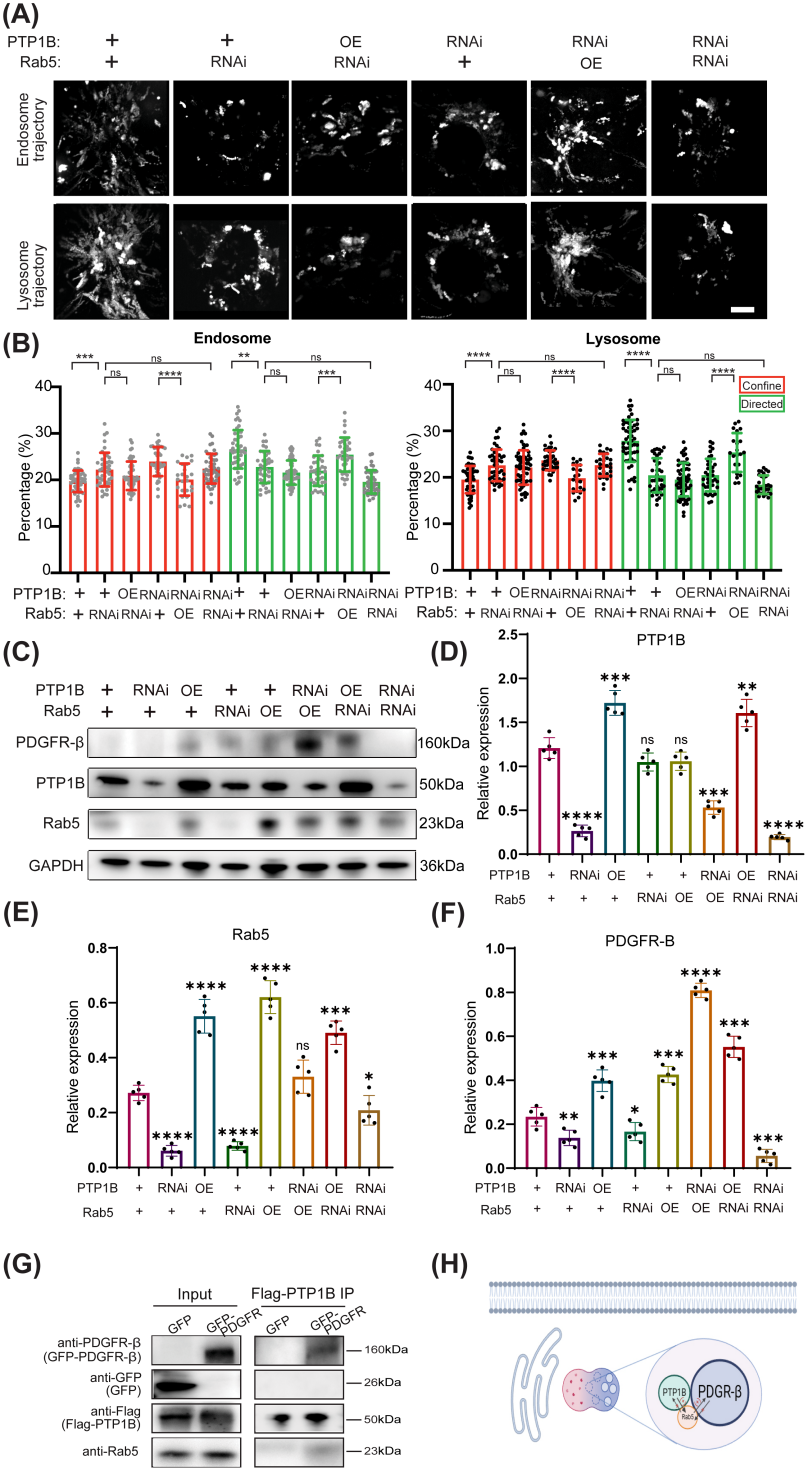
PTP1B modulates PDGFR‐β signaling via Rab5‐mediated endocytosis. OE, Overexpression; RNAi, RNA interference. (A) Endosome and lysosome trajectories of cos7 cells with the treatments of PTP1B knockdown or overexpression, and Rab5 knockdown or overexpression. (B) Quantitative analysis of confined and directed modes of endosome and lysosome. (C) Western blot analyses of PDGFR‐β, PTP1B, and Rab5. (D) The relative level of PTP1B/GAPDH (*n* = 5). (E) The relative level of Rab5/GAPDH (*n* = 5). (F) The relative level of PDGFR‐β/GAPDH (*n* = 5). (G) Western blotting images of Coimmunoprecipitation to test the interaction among PTP1B, Rab5, and PDGFR‐β. (H) Schematic diagram of relationship among PTP1B, Rab5, and PDGFR‐β on endocytosis system. Scar bar 10 μm. *p < 0.05, **p < 0.01, ***p < 0.001, ****p < 0.0001, and ^ns^ *p* > 0.05.

PTP1B is required not only to complete the recycling process of PDGFR‐β but also to transduce signals to downstream effectors. Low‐density lipoprotein receptor‐related protein 1 (LRP1) forms a signaling complex with PDGFR‐β in endosomes and regulates activation of the MAPK pathway. And thymosin β4 protects against aortic aneurysm via endocytic regulation of growth factor signaling. We, therefore, focus on the role of PTP1B in the endocytosis of PDGFR‐β by modulating the activity or expression of Rab5, to determine if this may, at least in part, explain the effects of the PTP1B/Rab5 axis on PDGFR‐β signaling. Compared to Rab5 RNAi treatment only, no obvious rescue nor enhancement of dynamic endocytosis was found in Rab5 shRNA RNAi cells upon overexpressing PDGFR‐β or further PTP1B inhibition (Figure 4A,B). However, overexpressing Rab5 restores the slowed‐down phenotype of PTP1B inhibition, suggesting that Rab5 levels determine the dynamics of endocytosis, and PTP1B is required for Rab5‐dependent endocytosis.

In addition, we used western blotting to clarify whether PTP1B modulates PDGFR‐β expression through Rab5 at protein level. The results showed that the expression of PDGFR‐β was regulated via PTP1B/Rab5 signaling pathway (Figure 4C). Overexpression of PTP1B or Rab5 restores the expression of PDGFR‐β upon the Rab5 inhibition or PTP1B inhibition, respectively (Figure 4C–F). The above results of western blotting suggested that PDGFR‐β signaling was modulated through PTP1B/Rab5 signaling pathway (Figure 4H). Immunoprecipitation (IP) was used to further explore the interaction among PTP1B, Rab5, and PDGFR‐β, and the results suggested that PTP1B interacted with Rab5 and PDGFR‐β (Figure 4G).

### 3.5 Increased PTP1B Expression Leads to cells' Apoptosis, Suppresses Carotid SMC Migration and Proliferation

**FIGURE 5:**
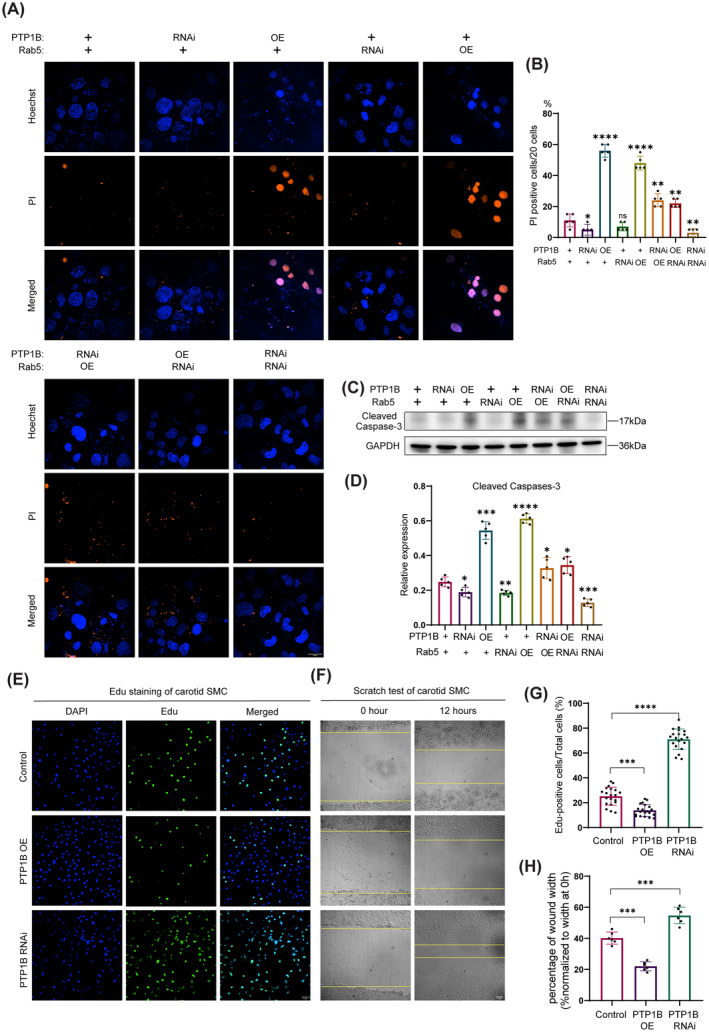
Increased PTP1B expression leads to cells' apoptosis, and suppresses carotid SMC migration and proliferation. RNAi: RNA interference. OE, Overexpression; PI, Propidium Iodide. Edu: 5‐ethynyl‐2′‐deoxyuridine. (A) Apoptosis was assessed by PI staining of cos7 cells treated with knockdown or overexpression of PTP1B and Rab5, respectively. Scar bar 20 μm. (B) Quantitative analysis of PI‐positive cells among 20 cells. (C) Western blot analyses of Cleaved Caspase‐3 of cos7 cells treated with knockdown or overexpression of PTP1B and Rab5, respectively. (D) The relative level of Cleaved Caspase‐3/GAPDH (*n* = 5). (E) Edu staining of carotid SMC treated with knockdown or overexpression of PTP1B. Scar bar 10 μm. (F) Scratch test of carotid SMC treated with knockdown or overexpression of PTP1B. Scar bar 10 μm. (G) Quantitative analysis of Edu‐positive cells. (H) Quantitative analysis of wound width. *p < 0.05, **p < 0.01, ***p < 0.001, ****p < 0.0001, and ^ns^ *p* > 0.05.

Cell apoptosis leads to inflammation and necrotic core formation, which results in plaque instability [17]. Therefore, we tested the impact of PTP1B/Rab5 signaling pathway on apoptosis using propidium iodide (PI) staining. The results showed that increased expression of PTP1B and Rab5 facilitated apoptosis, while downregulation of PTP1B protected cells from apoptosis. Enhancement of apoptosis was found in PTP1B or Rab5 shRNA RNAi cells upon overexpressing Rab5 or PTP1B, respectively. Double knockdown PTP1B and Rab5 significantly decreased the apoptotic level of cells (Figure 5A,B). Cleaved‐Caspase 3 is an activated form of the apoptotic effector protein. Similar results of protein level of Cleaved‐Caspase 3 were also detected according to the PI results (Figure 5C,D). Vulnerable plaque has a lower portion of VSMCs and thinner fibrous cap than stable lesions [18, 19]. We also tested the carotid VSMCs migration and proliferation using Edu staining and scratch wound test. The results confirmed that knockdown PTP1B significantly increased carotid SMC migration in the scratch test and proliferation in the Edu staining. When overexpressed the PTP1B, the results were opposite (Figure 5E–H). Overall, these results suggested that increased PTP1B expression led to cells' apoptosis, and suppressed carotid VSMCs migration and proliferation.

### 3.6 PTP1B Deficiency Decreases Mouse Atherosclerosis

**FIGURE 6:**
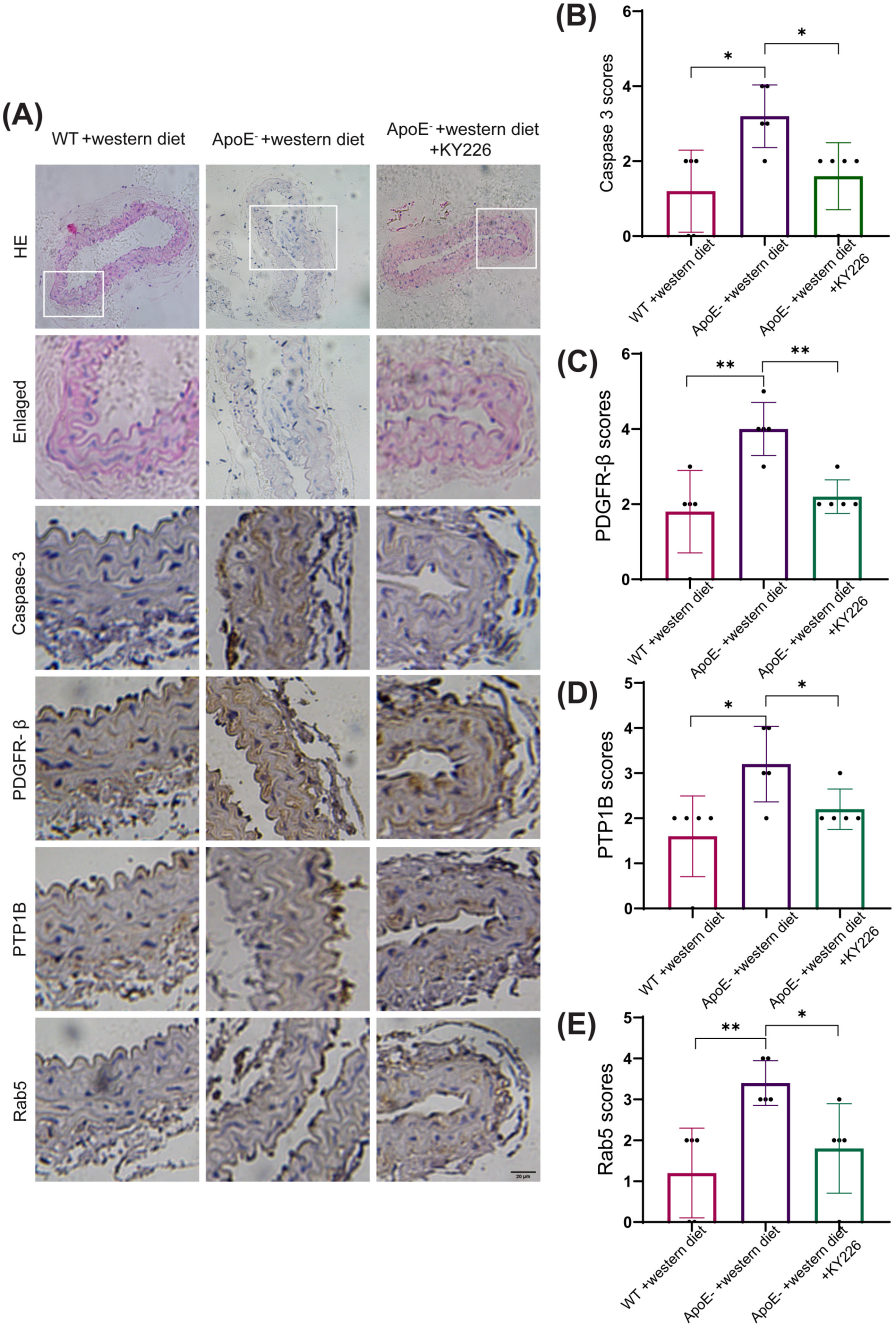
PTP1B deficiency decreases mouse atherosclerosis. HE, Hematoxylin–Eosin; WT, Wide type. (A) HE and immunohistochemical staining for Caspase‐3, PDGFR‐β, PTP1B, and Rab5 in representative mouse carotid arteries from Wildtype +western diet, ApoE^−^ + western diet and ApoE^−^ + western diet +KY226. (B–E) Quantitative analysis of immunohistochemical staining scores for Caspase‐3, PDGFR‐β, PTP1B, and Rab5 in mouse carotid arteries. *p < 0.05, **p < 0.01.

KY226 (inhibitor of PTP1B) was administered to ApoE^−/−^ mice with atherosclerosis to explore whether PTP1B deficiency decreases the level of atherosclerosis in vivo. The HE staining results showed that atherosclerosis was formed on carotid artery of ApoE^−/−^ mice fed with a western diet after 3 months, compared to WT mice (Figure 6A). The atherosclerotic level of carotid artery of ApoE^−/−^ mice fed with western diet and KY266 was decreased compared to ApoE^−/−^ mice fed with western diet (Figure 6A). Carotid artery from three groups of mice was stained with Caspase‐3, PDGFR‐β, PTP1B, and Rab5, and analyzed by immunohistochemistry. The results showed that the expressions of Caspase‐3, PDGFR‐β, PTP1B, and Rab5 were significantly increased in the group of ApoE^−/−^ mice fed with a western diet. However, the expressions of above proteins were significantly decreased in the group of ApoE^−/−^ mice fed with a western diet and KY226 (Figure 6A–E). These results suggested that PTP1B deficiency suppressed the carotid artery apoptosis of mouse and decreased the level of mouse atherosclerosis.

## 4 Discussion

This study investigated the expression pattern of PTP1B in atherosclerotic carotid plaque and explored the subcellular regulating mechanism of PTP1B to endo‐lysosome degradation of PDGFR by histological and biological assays. Up‐regulation of PTP1B disrupted the sustained and balanced motility of the endocytosis system, which resulted in the accumulation of PDGFR‐β. Further, PTP1B modulated PDGFR‐β signaling via Rab5‐mediated endocytosis. The accumulation of PDGFR‐β deteriorated carotid plaque apoptosis and therefore promoted plaque vulnerability. The above results may suggest PTP1B as a potential therapeutic target in stabilizing carotid plaque at subcellular level.

PTP1B is an important member of protein tyrosine phosphatases (PTPs) superfamily and is involved in many molecular mechanisms. During the previous studies, researches about PTP1B mainly focused on diabetes mellitus, because PTP1B is a negative insulin signaling pathway regulator; hence inhibiting PTP1B increases insulin sensitivity and effectively controls diabetes [20]. In addition, the role of PTP1B involved in the process of atherosclerosis is increasingly gaining attention. PTPs are critically involved as negative regulators for VSMCs growth and migration promoted by tyrosine phosphorylation, which is crucial for the pathogenesis of atherosclerosis [21]. In mice, myeloid PTP1B deficiency protects against atherosclerotic plaque formation in the ApoE^−/−^ mouse model of atherosclerosis [22]. In our study, we found that increased PTP1B expression leads to apoptosis, suppresses carotid VSMCs migration and proliferation, and promotes the vulnerability of carotid plaque. In addition, inhibition of PTP1B in mice could decrease the level of atherosclerosis. Therefore, PTP1B may be a potential target for reducing the risk of atherosclerosis.

PDGF‐ PDGFR‐β signaling is reported to be associated with vascular diseases, and plays a key role in VSMCs migration and proliferation during atherosclerosis [23, 24]. A previous study found that increased PDGFR‐β leads to chemokine secretion and leukocyte accumulation, and promotes advanced plaque formation. However, inhibition of PDGFR‐β activated transcription factor STATA in VSMCs relieves arterial inflammation and reduces plaque burden [11]. With regard to the relationship between PDGFR‐β and apoptosis, there is controversy among the studies, and this part of the research mainly focuses on the field of cancer. The positive rate of PDGFBB and/or PDGFR‐β was higher in meningioma, and apoptotic cells were also increased. However, the relationship between PDGFR‐β and apoptosis has not been deeply explored. Some studies considered that the association of PDGFR‐β leading to apoptosis may be an indirect process. Abnormal accumulation of PDGFR‐β would activate a series of downstream pathways, like MAPK and PI3K, and then these pathways induce the apoptotic signal [25, 26]. In addition, the abnormal activation of PDGFR‐β may be related to the DNA damage repair process of cells, and the abnormal DNA damage repair may lead to the initiation of apoptosis [27].

Receptor‐mediated endocytosis involves the internalization of ligands interacting with a cell surface receptor through a clathrin‐coated vesicle, followed by a sequence of processing steps [28]. Stable endocytic motility regulates compartmentalization and protein abundance within cells and is involved in many signaling pathways [29]. Endocytosis is determined as a key step in the regulation of the activity of PDGFR‐β. The PDGFR‐β recycling is regulated by Rab5‐mediated endosome transportation. In our study, we found that loss of Rab5 decreases the expression of PDGFR‐β which is responsible for the endocytic motility. We also explored the regulatory relationship between Rab5 and PDGFR‐β at the protein level. The increase in Rab5 accelerates the PDGFR‐β recycling, resulting in the increase in PDGFR‐β. In addition, we further found that PTP1B modulated PDGFR‐β signaling via Rab5‐mediated endocytosis. The protein tyrosine phosphatase (PTP) family is a critical regulator of a variety of cellular signaling pathways, including the PDGFR [30]. The previous findings indicated that PTP1B plays a dual role, exerting both positive and negative regulatory effects on PDGFR signaling [31]. The excessive expression of PTP1B effectively nullified the PDGF‐triggered tyrosyl phosphorylation of PDGFR‐β in insulin‐sensitive cells [32]. This factor potentially results in the accumulation of PDGFR‐β. However, the specific mechanism remains unclear. PTP1B‐Rab5‐PDGFR‐β signal axis may represent a potential regulating mechanism, which is expected to influence the progression of carotid plaque. However, since some experiments are more difficult to perform with human carotid artery smooth muscle cells and COS7 cells are easier to handle and commonly used in experimental research, we chose the COS7 cell line for certain experiments in this study. This introduces certain limitations to our research.

## 5 Conclusion

In conclusion, the presence of PTP1B in carotid plaque tissue exhibited a positive correlation with plaque vulnerability. Increased PTP1B levels disrupted the endo‐lysosomal interaction, resulting in the accumulation of PDGFR‐β and subsequent elevation in apoptosis, thereby fostering plaque vulnerability. The PTP1B‐Rab5‐PDGFR‐β axis appears to play a pivotal role in this biological regulation. Notably, this study unveiled, for the first time, the association between PTP1B and endocytic motility, presenting novel evidence supporting the potential use of PTP1B inhibitors in the treatment of vulnerable carotid plaques.

## Author Contributions

W.L. and L.J. designed and supervised all experiments of this study. X.Z., R.X., and T.W. performed the histological and biological studies. J.L., S.C., Z.X., and G.Y. analyzed and interpreted the experimental data. Y.S. and X.L. contributed to data analysis. All authors were involved in writing the manuscript, and all read approved the final manuscript.

## Conflicts of Interest

The authors declare no conflicts of interest.

## Supporting information


**Figure S1.** Efficiency of knockdown and overexpression of PTP1B, PDGFR‐β, and Rab5. OE, overexpression; RNAi, RNA interference. (A) The efficiency of knockdown and overexpression of PTP1B were examined by Western blot. (B) The efficiency of knockdown and overexpression of PDGFR‐β were examined by Western blot. (C) The efficiency of knockdown and overexpression of Rab5 were examined by Western blot.

## Data Availability

The data that support the findings of this study are available from the corresponding author upon reasonable request.
