# Supplementary figures and images for: PTP1B Modulates Carotid Plaque Vulnerability in Atherosclerosis Through Rab5‐PDGFRβ‐Mediated Endocytosis Disruption and Apoptosis
Source: CNS Neurosci Ther. 2024 Nov 8;30(11):e70071. doi: 10.1111/cns.70071 (PMC11549062; doi:10.1111/cns.70071)

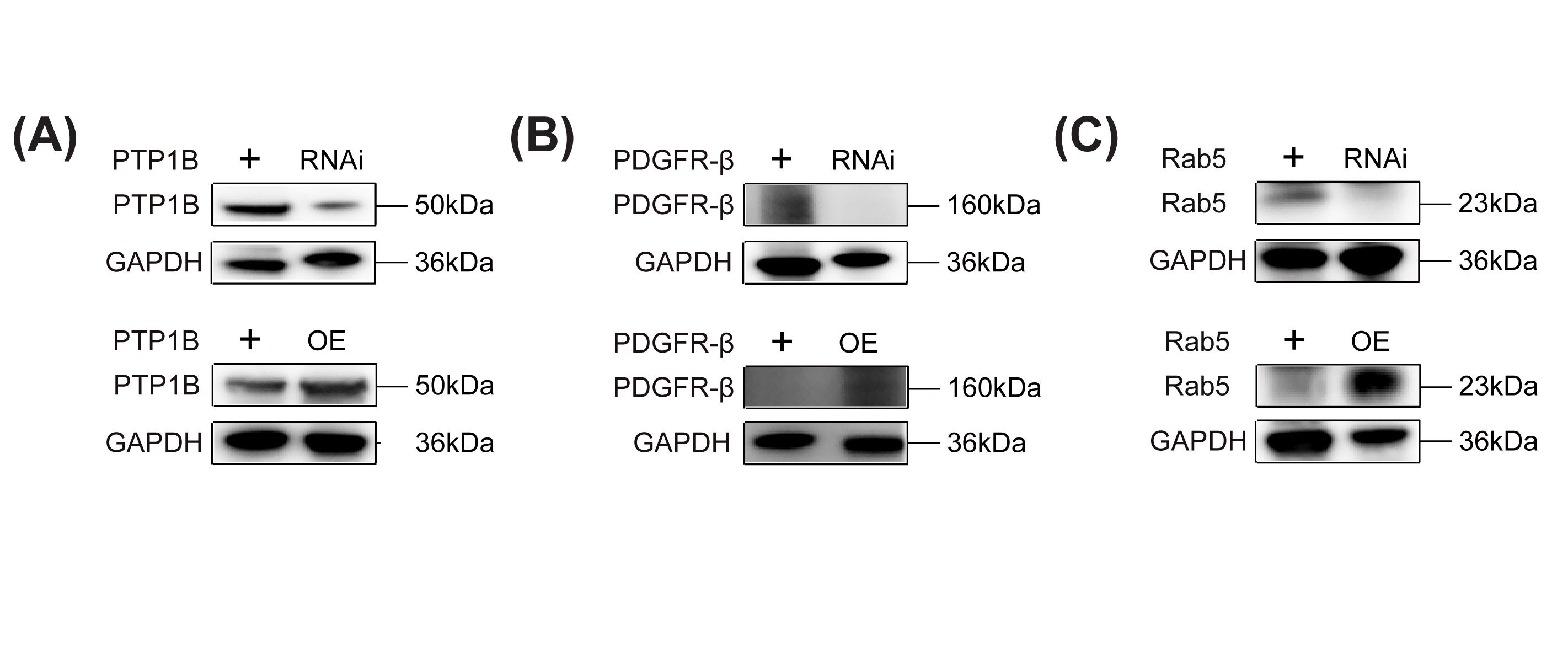

Supplement: Supplementary file 1 — Figure S1. Efficiency of knockdown and overexpression of PTP1B, PDGFR‐β, and Rab5. OE, overexpression; RNAi, RNA interference. (A) The efficiency of knockdown and overexpression of PTP1B were examined by Western blot. (B) The efficiency of knockdown and overexpression of PDGFR‐β were examined by Western blot. (C) The efficiency of knockdown and overexpression of Rab5 were examined by Western blot. [file CNS-30-e70071-s001.tif]
